# Successful treatment of a multifocal comminuted open fracture of humerus with severe soft tissue defect: a case report

**DOI:** 10.1186/s13256-023-03989-9

**Published:** 2023-06-26

**Authors:** Hasan Barati, Sina Afzal

**Affiliations:** Department of Orthopedic surgery, School of Medicine, Imam Hossein Hospital, Shahid Beheshti University of Medical Sciences, Tehran, Iran

**Keywords:** Open fracture, Humerus, Soft tissue defect, Skin graft, Vacuum dressing, Elastic intramedullary nail, External fixation, Case report

## Abstract

**Background:**

Choosing the appropriate treatment approach for a multifocal comminuted open fracture of humerus with severe soft tissue defect is a challenging issue, which could be interesting for every orthopedic surgeon especially for those working in the trauma centers.

**Case presentation:**

This study described an innovative approach using titanium elastic nailing to treat a multifocal comminuted open fracture of humerus with severe soft tissue defect. In this study, we report a 40-year-old Persian female patient in whom the treatment achieved complete fracture union and skin graft healing by elastic medullary nailing, vacuum dressing, and skin grafting.

**Conclusions:**

Elastic medullary nailing is a viable option for reconstruction of simultaneous comminuted fracture and soft tissue defect.

## Background

Ipsilateral multifocal humeral fracture is an infrequent injury [1]. In addition, head-split and anatomical neck humeral fractures are extremely rare [2, 3]. Coincidence of highly comminuted proximal and diaphyseal fracture makes these conditions even more complicated and challenging [4]. Concomitance of severe soft tissue defect requiring soft tissue reconstruction with aforementioned injuries is scarce in literature [5, 6]. Choosing the best treatment strategy for a multifocal comminuted open fracture of humerus associated with severe soft tissue defect and articular involvement is challenging, which could come up for every orthopedic surgeon specially for those working in trauma centers. In this study, we reported a patient with multifocal comminuted open fracture of humerus with severe soft tissue defect who was treated with and innovative approach using elastic medullary nailing and skin grafting to repair the humerus and soft tissue injury. To the best of our knowledge, no similar patient with such complex injuries has been described and treated with this approach in the literature, and the reported case and treatment approach in this study could help future cases and surgeons to achieve successful outcomes.

## Case presentation

**Fig. 1:**
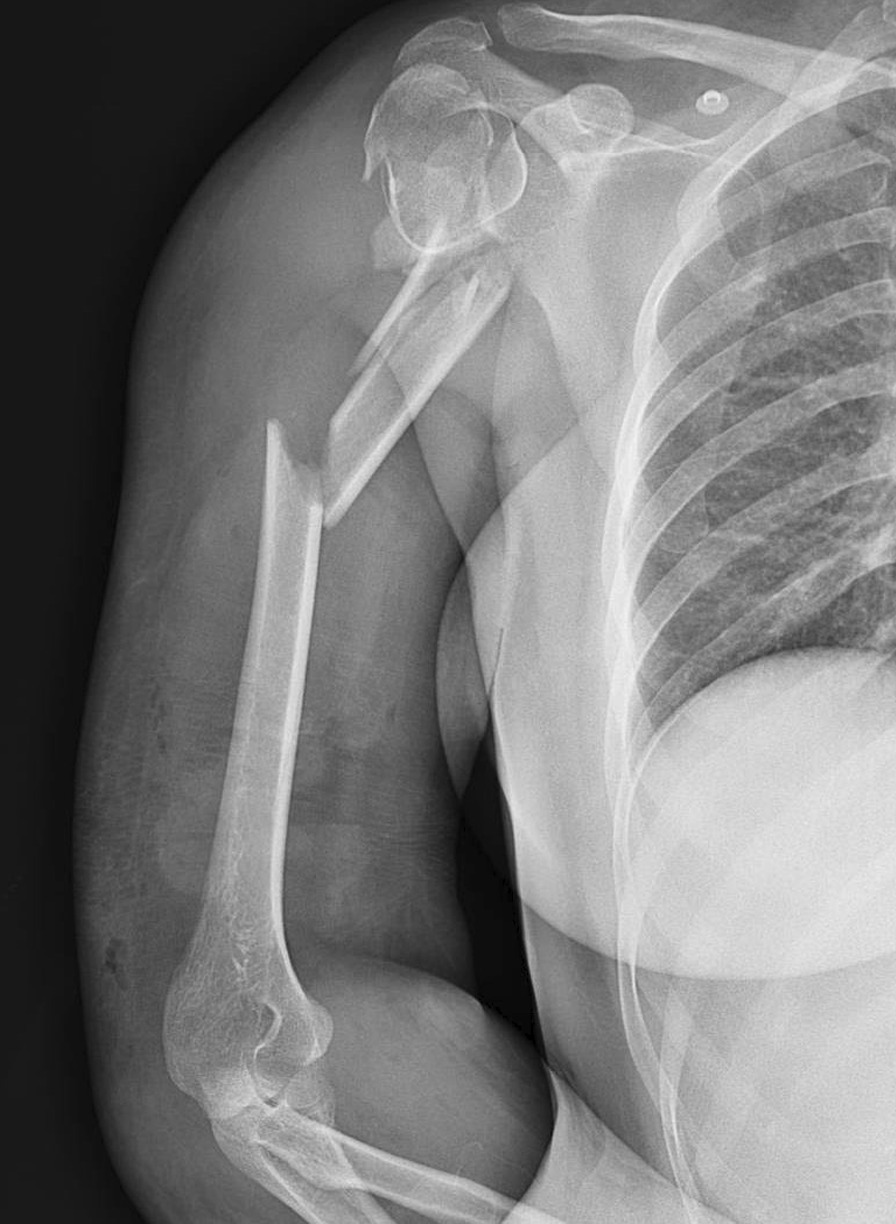
Concomitant 3-part head-splitting proximal humerus and comminuted humeral shaft fractures in preoperative X-ray

A 40-year-old Persian female patient was referred to a tertiary trauma center one day after the motor-vehicle-accident. Patient had no significant past medical history. Following the primary wound management, a digital X-ray was obtained from her shoulder that demonstrated a concomitant 3-part head-splitting proximal humerus and comminuted humeral shaft fractures (Fig. 1). The patient was referred to our center for surgical treatment. Initial evaluations in our center revealed no other associated major injury, and the neurovascular assessments were normal. The wound at the location of injury sized about 14 cm in length and 5 cm in width, located on anteromedial aspect of upper portion of the arm, showing a Gustilo-Anderson type of IIIB fracture. A long arm splint was applied after wound irrigation and dressing.

Surgery was performed in semi-sitting position on the following day. First, the wound was irrigated with low pressure pulsatile manner with more than 6 L of normal saline serum, followed by the debridement of the unviable tissues. Through a deltopectoral approach, reduction of greater tubercle, anatomical neck and head-split component (about 20% of head) attained and were fixed by 3 screws. Afterwards, two small incisions were made over both epicondyles of humerus, and after awl insertion, fluoroscopic guided closed reduction of humerus was achieved by longitudinal traction, external rotation, and abduction. Two titanium elastic nail (TEN) were inserted with rotational forces and anchored to the subchondral bone since the humeral head had a fracture. The post-operative neurovascular tests were normal. The patient was discharged and advised to use a 10-day vacuum dressing. Following the confirmation of absence of any infection and growth of the granulation tissue, patient was readmitted for skin graft. After 5 days, patient was discharged with necessary post-operative instructions after graft site healing assurance.

**Fig. 2:**
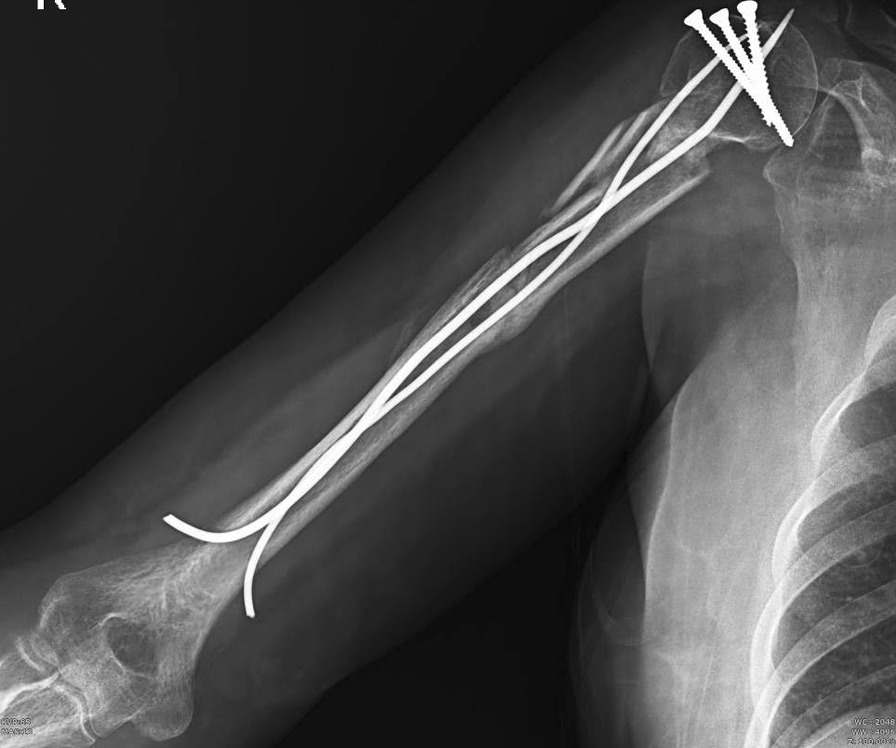
Anteroposterior X-ray showing partial union in second postoperative month

**Fig. 3:**
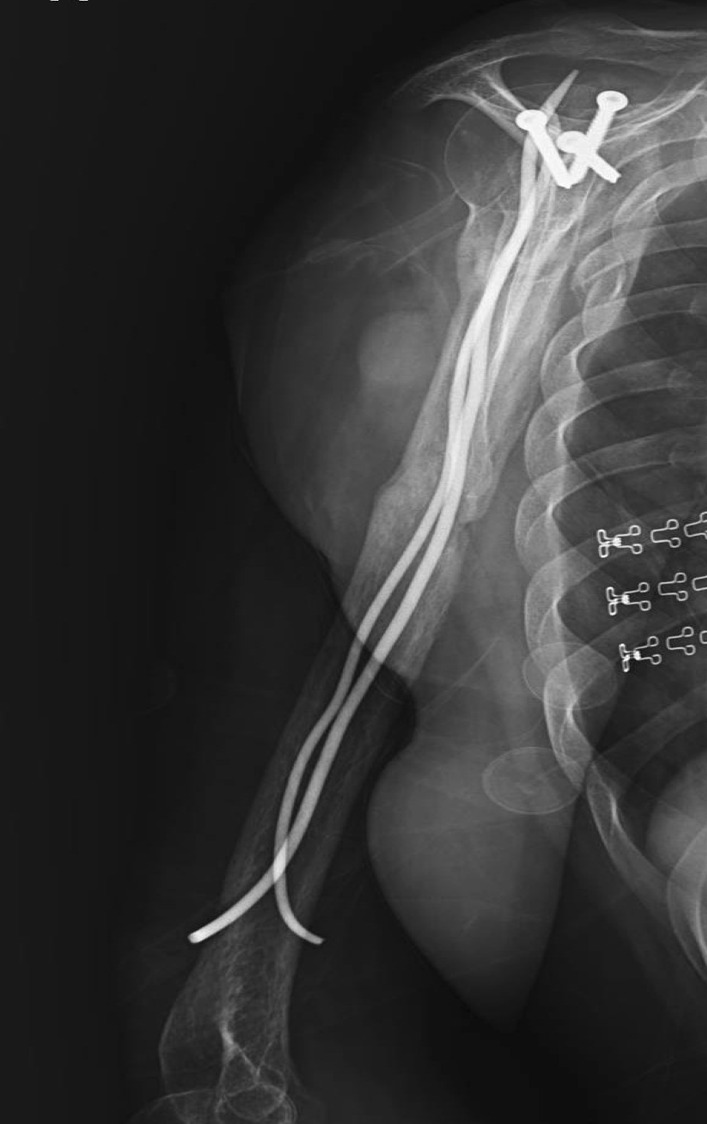
Lateral X-ray showing partial union in second postoperative month

**Fig. 4:**
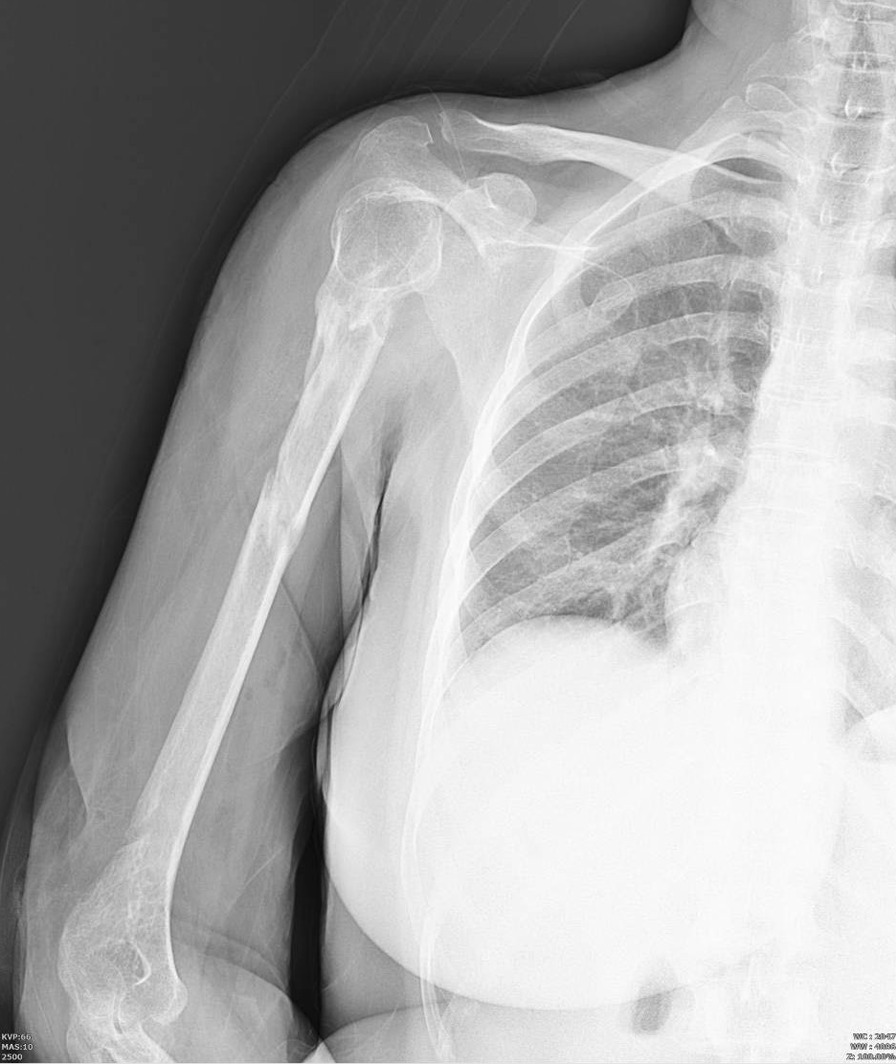
Complete union in 6th month postoperative

After 20 days while full healing of the donor and graft sites were achieved, the sutures and splint were removed. Splint was replaced with Sarmiento brace and the elbow movements were initiated. Two months after the trauma, full elbow range of motion (ROM) was achieved. A partial union (consolidation) was found on the X-ray unexpectedly, and considering the presence of comminution and fixation with elastic nails which is a non-rigid fixation, we continued the splint until the partial union was achieved and then removed it so that the patient could go through the recovery period with less discomfort. Thereafter, the brace was discontinued and passive pendulum shoulder exercise commenced (Figs. 2, 3). After 6 months, a near total union was observed in X-ray; therefore, all screws and TENs were removed. Thereafter physiotherapy for patient was done for 20 sessions. At the end of the 7th month of the follow-up period, complete union of the fracture was achieved (Fig. 4.), with no signs of infection and full elbow ROM. However, a small limitation of abduction and forward flexion in terminal ROM were detected and the assessed disabilities of the arm, shoulder and hand (DASH) score was10.

## Discussion and conclusion

According to the available literature, several options have been introduced for humeral fractures; including, closed reduction and splinting, closed reduction and intramedullary nailing (both rigid and elastic), open reduction and internal fixation with various plates, minimally invasive percutaneous plate osteosynthesis (MIPPO), and external fixation and arthroplasty [7, 8]. Arthroplasty is an alternative treatment option for 3-parted and displaced anatomical neck fracture, but regarding to the patient’s age and massive soft tissue defect, we preferred to try an osteosynthesis method first [9]. Being considered as a relative indication, fixation is recommended in both comminuted and open fractures [7, 10]. Therefore, we chose a fixation strategy for the management of this patient.

The surgical approach mostly used in humeral fractures is open reduction and internal fixation (ORIF) with plate, but considering the comminution and the risk of non-union in our patient, this method was not applicable. Other surgical strategy which could be applied to treat the comminuted fractures is MIPPO, while the reason we did not use this method was the higher risk of infection in presence of extreme soft tissue defect that could jeopardize the outcomes of surgical repair of the fracture [11].

External fixation is a viable option for patients with multiple trauma who are clinically unstable and those with severe soft tissue damage; however, Zhang *et al*. described this method for stable patients with closed fractures [12]. Weerasuriya *et al*. in 2011 reported a case with shattered floating shoulder and extreme soft tissue damage who was treated by external fixator to fix the humerus to iliac crest as a limb salvage treatment. The aforementioned technique was employed for the purpose of salvaging shoulder injuries that had been shattered in patients who possessed a functional hand and an intact neurovascular bundle, with the aim of averting shoulder disarticulation [13].

By using proper antibiotic therapy and wound management, several studies have demonstrated that the risk of infection in intramedullary nailing and external fixation has no difference [14, 15]. External fixation could be used as a treatment option for our patient, but the treatment strategy we applied had several advantages; including, better wound management, absence of pin tract infection, and patient comfort [16]. On the other hand, the safe zone of the proximal pin of external fixator was comminuted in our patient that could fail the successful use of an external fixator [16].

Although both rigid and elastic nailing material could be applied in this case, rigid nailing was not an suitable option because its proximal screws were in line with proximal fracture site and could not obtain an acceptable grip [17]. As another advantage of the used method, the risk of nerve palsy was lower compared to the other surgical methods specially open procedures [7]. Concluding, we used elastic nailing to manage this case and according to the successful results of our approach in this case, we recommend further application of elastic nailing in similar complex injuries.

Although the surgical intervention in severe injuries like those including the musculoskeletal system is essential to save the patients, the use of unnecessary and expensive materials for orthopedic surgeries can cause financial difficulties for patients and lead to the wastage of medical resources [18]. In addition, it is important to select the biomaterials used for surgeries correctly, and instead of using unnecessary or expensive materials, it is possible to perform the surgery effectively and safely by making the right material selection [19]. As another surgical principle in the orthopedics field, the tissues should be damaged as little as possible during surgeries, and the least invasive interventions should be performed, so the patients' recovery process would be faster and less painful in this way [20]. Putting together, it is important to respect the tissue and use the right materials for surgery to facilitate the patients' recovery process and use the medical resources more effectively.

In conclusion, in concomitant comminuted proximal and diaphyseal humeral shaft fractures which need further soft tissue surgeries, application of TENs can serve as a viable option to achieve acceptable outcomes of both bony structures’ fracture and soft tissue injuries.

## Data Availability

The material presented in this study are available from the corresponding author on a reasonable request.

## Abbreviations

TEN: Titanium elastic nail
ROM: Range of motion
DASH: Disabilities of the arm, shoulder and hand
